# Whole Genome Sequencing and Antimicrobial Resistance of *Staphylococcus aureus* from Surgical Site Infections in Ghana

**DOI:** 10.3390/pathogens10020196

**Published:** 2021-02-12

**Authors:** Beverly Egyir, Jeannette Bentum, Naiki Attram, Anne Fox, Noah Obeng-Nkrumah, Labi Appiah-Korang, Eric Behene, Selassie Kumordjie, Clara Yeboah, Bright Agbodzi, Ronald Essah Bentil, Rhodalyn Tagoe, Blessing Kofi Adu Tabi, Felicia Owusu, Nicholas T. K. D. Dayie, Eric S. Donkor, Josephine Nsaful, Kwaku Asah-Opoku, Edward Nyarko, Edward Asumanu, Anders Rhod Larsen, David M. Wolfe, Andrew G. Letizia

**Affiliations:** 1 Bacteriology Department, Noguchi Memorial Institute for Medical Research, University of Ghana, Accra 00233, Ghana; bentum.jeannette@yahoo.com (J.B.); rhodalyntagoe1310@gmail.com (R.T.); kofitabi39@gmail.com (B.K.A.T.); owusufelicia90@gmail.com (F.O.); 2 Naval Medical Research Unit—Three, Ghana Detachment, Accra 00233, Ghana; NPuplampu@noguchi.ug.edu.gh (N.A.); Ghana; afox@camris.com (A.F.); ebehene@gmail.com (E.B.); majozk@yahoo.co.uk (S.K.); sayhi2clara@yahoo.com (C.Y.); bright.agbodzi@gmail.com (B.A.); ronbentil@hotmail.com (R.E.B.); davidwolfe@gmail.com (D.M.W.); andrew.g.letizia.mil@mail.mil (A.G.L.); 3 Department of Medical Laboratory Sciences, School of Biomedical and Allied Health Sciences, University of Ghana, Accra 00233, Ghana; nobeng-nkrumah@ug.edu.gh; 4 Department of Microbiology, Korle-Bu Teaching Hospital, Accra 00233, Ghana; guylabi2@gmail.com; 5 Department of Medical Microbiology, University of Ghana Medical School, University of Ghana, Accra 00233, Ghana; nicholasdayie@yahoo.com (N.T.K.D.D.); ericsdon@hotmail.com (E.S.D.); 6 Department of Surgery, Korle-bu Teaching Hospital, Accra 00233, Ghana; josco19@yahoo.com; 7 Department of Obstetrics and Gynaecology, University of Ghana Medical School, University of Ghana, Accra 00233, Ghana; kasahopoku@yahoo.com; 8 37 Military Hospital, Accra 00233, Ghana; eonyarko@yahoo.co.uk (E.N.); easumanu@yahoo.com (E.A.); 9 Statens Serum Institut, Department of Bacteria, Parasites and Fungi, Reference Laboratory for Antimicrobial Resistance, Artillerivej 5, DK-2300 Copenhagen, Denmark; arl@ssi.dk

**Keywords:** surgical site infections, MRSA, whole-genome sequencing, Africa

## Abstract

*Staphylococcus aureus* (*S. aureus)* is a common cause of surgical site infections (SSIs) globally. Data on the occurrence of methicillin-susceptible *S. aureus* (MSSA) as well as methicillin-resistant *S. aureus* (MRSA) among patients with surgical site infections (SSIs) in sub-Saharan African are scarce. We characterized *S. aureus* from SSIs in Ghana using molecular methods and antimicrobial susceptibility testing (AST). Wound swabs or aspirate samples were collected from subjects with SSIs. *S. aureus* was identified by matrix-assisted laser desorption ionization–time of flight mass spectrometry (MALDI-TOF-MS); AST was performed by Kirby-Bauer disk diffusion, and results were interpreted according to the Clinical and Laboratory Standards Institute (CLSI) guideline. Detection of *spa, mecA*, and *pvl* genes was performed by polymerase chain reaction (PCR). Whole-genome sequencing (WGS) was done using the Illumina MiSeq platform. Samples were collected from 112 subjects, with 13 *S. aureus* isolates recovered. Of these, 92% were sensitive to co-trimoxazole, 77% to clindamycin, and 54% to erythromycin. Multi-drug resistance was detected in 5 (38%) isolates. The four *mecA* gene-positive MRSA isolates detected belonged to ST152 (*n* = 3) and ST5 (*n* = 1). In total, 62% of the isolates were positive for the Panton-Valentine leukocidin (*pvl*) toxin gene. This study reports, for the first time, a *pvl*-positive ST152-t355 MRSA clone from SSIs in Ghana. The occurrence of multi-drug-resistant *S. aureus* epidemic clones suggests that continuous surveillance is required to monitor the spread and resistance trends of *S. aureus* in hospital settings in the country.

## 1. Introduction

Surgical site infections (SSIs) are infections that occur at the surgical site within 30 or 90 days and up to a year (if there is an implant) of a surgical procedure [1]. Globally, SSIs constitute 14–33% of hospital-acquired infections [2,3,4,5,6], and approximately 2–5% of surgical patients have been estimated to develop an infection [2]. SSIs result in delayed wound healing, prolonged hospitalization, increased readmission rates, and increased healthcare cost, as well as increased morbidity and mortality [4,7,8,9,10].

*Staphylococcus aureus* (*S. aureus*) is a frequent cause of SSI [11,12,13]. Among *S. aureus* strains, the prevalence of methicillin resistant *S. aureus* (MRSA) is rising on the African continent [14,15,16,17]. This is of major concern due to the multi-drug-resistant nature of MRSA and the limited therapeutic options available to treat infected patients.

In Ghana, SSIs are the most common healthcare-associated infections and account for up to 33% of all hospital-acquired infections [5]. Although *S. aureus*, particularly MRSA, in SSI can have severe adverse prognostic implications, scant epidemiologic information exists, including information on its occurrence, antimicrobial resistance and molecular characteristics. In this study, we describe the antimicrobial resistance patterns and molecular characteristics of *S. aureus* recovered from subjects with SSIs at two hospitals in Accra, Ghana.

## 2. Results

Of the 112 subjects, 56 (50%) and 56 (50%) were enrolled at the 37 Military Hospital (37-MH) and Korle-Bu Teaching Hospital (KBTH), respectively. Subjects recruited at the 37-MH consisted of 28 (50%) males; those from KBTH comprised 50 (89.3%) females. Table 1 describes the population sampled at the two hospitals. Data collected on antibiotic use before and after surgery indicate that metronidazole, amoxicillin/clavulanic acid, clindamycin, and ciprofloxacin were frequently administered to subjects in both hospitals.

### 2.1. Proportions of Subjects Positive for Staphylococcus aureus and Antimicrobial Resistance

Culturing of one sample per patient showed bacterial growth (i.e., *S. aureus*, *K. pneumoniae, E. coli*, and *P. aeruginosa*) in 70 (62.5%) of the 112 samples. *S. aureus* was identified in 13 of 70 culture-positive samples (18.6%). Of the 13 *S. aureus* positive patients, two were co-infected with *E.coli,* two with *P. aeruginosa,* and five with *K. pneumoniae.*

**Figure 1:**
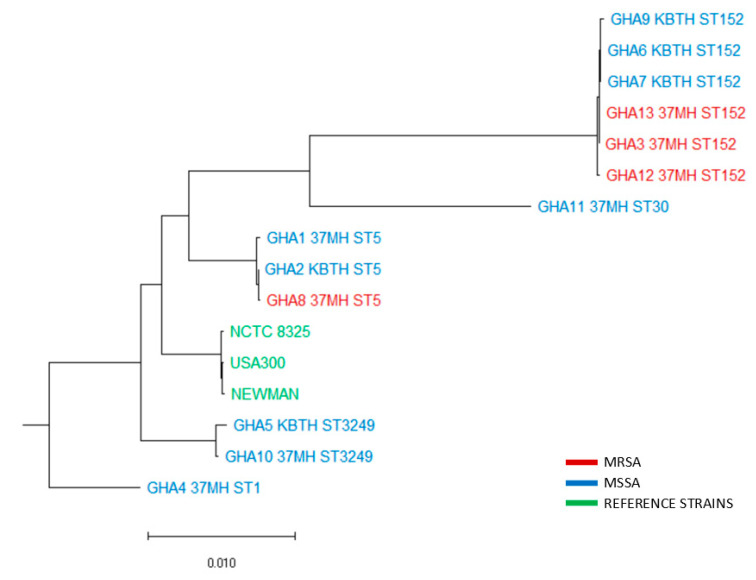
The whole-genome phylogeny of the 13 *S. aureus* isolates. The maximum likelihood phylogeny was constructed using RAxML v 4.0 and based on the GTR GAMMA nucleotide substitution model. The whole-genome alignment was done using Mauve v 1.1.1. The total number of nucleotide changes per sequence is reflected in the branch length. Three reference strains, USA300, Newman, and NCTC 8325, were included in the analysis. The sequences were named with strain names, hospital of isolation (37-MH: 37 Military Hospital; KBTH: Korle-Bu Teaching Hospital), and sequence type (ST). The sequences formed monophyletic clades based on their sequence types.

Antimicrobial susceptibility testing showed full susceptibility to gentamicin but resistance to penicillin (100%; 13/13), tetracycline (46%; 6/13), erythromycin (46%; 6/13), cefoxitin (31%; 4/13), clindamycin (23%; 3/13), and co-trimoxazole (8%; 1/13). Multi-drug resistance was detected in 5 (38%) isolates. The four cefoxitin-resistant (MRSA) isolates originated from 37-MH and were susceptible to vancomycin but resistant to clindamycin (50%; 2/4), erythromycin (75%; 3/4), and tetracycline (50%; 2/4). Whole-genome sequencing indicated that the three MRSA ST152 isolates were related despite differences in AST (Figure 1).

### 2.2. Whole-Genome Sequence Analysis

**Genome analysis indicated that the isolates belonged to five sequence types: ST152 (46.1**%; 6/13), ST5 (23.1%; 3/13), ST3249 (15.4%; 2/13), ST30 (7.7%; 1/13), and ST1 (7.7%; 1/13). The predominant *spa* type was t355 (*n* = 5). Eight (61.5%) of the 13 isolates were positive for the *lukF-PV* and *lukS-PV* genes encoding the PVL toxin. The *hlgB* (100%; 13/13), *hlgA* (92.3%; 12/13), and *hlgC* (38.5%; 5/13) toxin genes were also detected among the isolates. A single isolate was positive for the *tst* gene, which encodes toxic shock syndrome toxin-1. There were several enterotoxin genes detected, with *sei* (30.7%; 4/13) being the most prevalent among the isolates. Host immune evasion genes, *scn* (92.3%; 12/13) and *sak* (30.7%; 4/13), were detected; the predominant exoenzyme gene was *aur* (38.5%; 5/13). Other exoenzyme genes were *splA* (30.7%; 4/13), *splB* (23.1%; 3/13), and *splE* (7.8%; 1/13). The prevalent resistance genes were *blaZ* (100%; 13/13) and *tet* (K) (46%; 6/13). Four isolates were positive for the *mecA* gene, three of which belong to PVL-positive t355 (ST152) with SCC*mec* type IVa (2B) and one (1) PVL-negative t002 (ST5) with SCC*mec* type Vc. Figure 1 shows the whole-genome phylogeny of the *S. aureus* isolates. The characteristics of all 13 isolates, as well as the details of patients from which isolates were recovered, are shown in Table 2. The complete genome sequences have been deposited at Gene Bank with the following accession numbers: CP043911-CP043923. Table 3 shows the summary of whole-genome assemblies.

## 3. Discussion

This study provides insights into the phenotypic and molecular characteristics of *S. aureus* and MRSA recovered from subjects with SSIs in Ghana. The proportion (11.6%) of SSIs positive for *S. aureus* found in this study is similar to what has been found in other countries [18,19]. On the contrary, a previous study conducted in a private hospital in Ghana recorded a 54% *S. aureus* prevalence [20]. Of note, the CDC criteria used in the selection of the subjects in this study were not used in the previous studies.

*S. aureus* isolates in Africa are known to have high rates of resistance to erythromycin, tetracycline, and co-trimoxazole due to frequent prescription of these drugs [21]. Therefore, the high level of resistance to tetracycline was not surprising since its use is still high in Ghana at the primary healthcare level. On the contrary, sensitivity to clindamycin and co-trimoxazole was high, probably because newer drugs have led to less use of those drugs for common indications. Additionally, the majority of the isolates were susceptible to clindamycin; this indicates that clindamycin is still useful for staphylococcal treatment in Ghana. The presence of three lincosamide-resistant isolates, likely due to frequent use in hospitals, suggests that resistance is emerging, and prudent use of this antibiotic is imperative.

Vancomycin is the drug of choice for treating MRSA infections [22], although reduced susceptibility of MRSA to vancomycin has been reported [17,23]. In this study, all MRSA isolates were fully susceptible to vancomycin, which is consistent with Ghanaian reports from healthy carriers and other types of infections [24,25,26,27], as well as in other parts of Africa [28,29]. These findings are most likely related to the low availability of the drug and the subsequent scant use in Ghana [30]. We observed that one MRSA isolate resistant to amoxicillin clavulanic acid and another resistant to ciprofloxacin were recovered from two different patients who were given the same antimicrobial agent for post-surgical antimicrobial therapy (Table 2). There is, therefore, a possibility that the treatment may be ineffective in such patients.

Previous studies in Ghana used *spa* and MLST typing of *S. aureus* isolates [24,25,26,27], while in this study, WGS was used to characterize the isolates. The use of WGS has many advantages in light of giving precise data on antimicrobial resistance, typing for outbreak control, and surveillance, as well as toxin gene profiling. In this study, we also found an isolate resistant to chloramphenicol, a drug that was not tested by disk diffusion (Table 2). These findings will help improve infection control within the hospital setting in the future.

We found PVL-positive ST152 (t355) as the dominant clone in the collection of *S. aureus* recovered from SSI isolates. This clone is known to be circulating in Ghana and widely distributed in many African countries as well as in Europe [21,26,31,32,33,34]. In Ghana, PVL-positive t355 (ST152) MSSA is a common clone and has been previously reported [26]. However, ST152-MRSA has not been identified despite examining over 500 *S. aureus* isolates originating from more than 2000 carriage and clinical human samples [24,25,26,27]. Consequently, the novel MRSA clones identified in this study demonstrate the shifting resistance patterns and potential selective pressure due to antibiotic use. PVL-positive ST152-MRSA is a pandemic clone that has been associated with community-acquired MRSA in Central Europe and the Balkans [34,35,36]. Since PVL-MRSA is associated with significant infections in patients without any risk factors [37], this finding is of concern and could inform the prognosis of SSIs in Ghana.

Our study also identified other sequence types, including ST5, ST30, ST3249, and ST1. The sequence type ST5 has been previously reported in Ghana [26,33], and one of the two ST5-MSSA in this study carried the *pvl* gene, consistent with previous reports [26]. It has been documented that ST30 is the common sequence type in Australia, but it has also been described in Ghana [24,25,26,27]. In addition, studies in other countries like South Africa, USA, and Germany [38] have also reported ST30-MSSA. Although ST30 commonly carries the PVL toxin [21,24,25,26], the ST30-MSSA in this study did not carry the *pvl* gene. This finding contrasts with what has been previously reported in Ghana [24,25,26]. The present study also identified a sequence type, ST3249, which has only been previously identified in a burn unit in a hospital in Ghana [39].

Various virulence factors allow *S. aureus* to colonize its host and cause disease. The enterotoxin genes (*sea, sei, sem, sen, seo, sek, sep*), leukocidin genes (*E and D*), and γ toxin genes (*hlgA, hlgB and hlgC*) identified in this study allow invasion and damage of host tissues [40]. PVL, leukocidin (E and D), and γ toxins (*hlgA, hlgB* and *hlgC*) have been suggested to contribute to enhanced virulence, suggesting worse clinical outcomes. This makes it necessary to properly identify genes for the purpose of controlling and monitoring their spread and informing infection control measures. Additionally, some studies have reported the *tst* gene associated with certain MSSA lineages [41,42]. Similarly, the only positive *tst* gene isolate was MSSA, belonging to t127 (ST1). The *tst* gene encodes the toxic shock syndrome toxin (TSST-1). This toxin is known to be secreted by some *S. aureus* isolates and causes toxic shock syndrome, which is a life-threatening condition.

## 4. Materials and Methods

### 4.1. Study Design, Site, and Sampling Procedures

A hospital-based cross-sectional study of SSI was conducted between June and November 2018 at the 37 Military Hospital (37-MH) and Korle-Bu Teaching Hospital (KBTH) in Accra, Ghana. Subjects from 37-MH were enrolled from the Surgical Outpatient Department and inpatient General Surgery, Obstetrics and Gynaecology, Orthopaedic, and Trauma wards. Subjects from KBTH were recruited from Surgical, Orthopaedics, Maternity, Obstetrics and Gynaecology, Neurosurgery, and Paediatric Surgery wards.

In this study, patients who had undergone surgery, had developed infections, and met the CDC criteria for classification as SSI [1] were recruited into the study. Demographic data, such as age, sex, ward of admission, operation type, period of hospitalization, and antimicrobial therapy, were collected from the medical records of each patient.

Using a sterile cotton-tipped applicator or a syringe, a wound swab, fluid, or aspirate was aseptically collected at the time of recruitment from subjects who gave informed consent. The samples were transported to the Noguchi Memorial Institute for Medical Research, University of Ghana, for phenotypic and molecular analysis.

### 4.2. Isolation and Identification of S. aureus

Samples were cultured on blood agar (Oxoid, Basingstoke, Hants, UK) and mannitol salt agar (Oxoid, Basingstoke, Hants, UK) and incubated for 18–24 h at 37 °C. *S. aureus* was identified by colonial morphology, Gram stain characteristics, catalase test, and coagulase test (Rabbit plasma) and confirmed using MALDI-TOF-MS (Bruker, Billerica, MA, USA).

### 4.3. Antimicrobial Susceptibility Testing

Antimicrobial susceptibility testing was performed by the Kirby-Bauer disk diffusion method using cefoxitin (30 µg), tetracycline (30 µg), clindamycin (2 µg), erythromycin (15 µg), gentamicin (10 µg), and trimethoprim/sulfamethoxazole (1.25/23.75 µg) from Oxoid (Basingstoke, Hants, UK). The minimum inhibitory concentration to vancomycin was determined with E-test strips (Biomérieux, Marcy-l’Étoile, France). Disk diffusion results were interpreted using the Clinical and Laboratory Standards Institute (CLSI) (2018) guideline. All isolates with cefoxitin zones sizes ≤21 mm (at 33–35 °C ambient air incubation for 16–18 h) were considered presumptive MRSA [43] and confirmed by PCR detection of the *mecA* gene.

### 4.4. PCR Detection of spa, pvl, and mecA genes

Crude DNA was extracted from *S. aureus* isolates as described previously [44]. A multiplex PCR was performed to detect the *spa, pvl*, and *mecA* genes as described previously [45], with slight modifications. Cycling conditions for PCR and primers for all genes detected are listed in Table 4. Each PCR tube contained 12.5 µL of multiplex PCR Mastermix (Qiagen, Hilden, Germany), 2.5 µL of RNase-free water (Qiagen, Hilden, Germany), 8 µL of primer mix, and 2 µL of DNA template. DNA amplification was performed using an Eppendorf Mastercycler (Eppendorf, Hamburg, Germany). A 2% *w/v* agarose gel was used to analyze the amplified products. The expected band sizes for the various genes were *spa* (variable region: 200–600 bp), *mecA* (162 bp), and *pvl* (80 bp).

The concentration of each primer (forward and reverse) used was as follows: 0.18 µM for *spa*, 1 µM for *pvl*, and 0.45 µM for *mecA.* Cycling conditions: initial denaturation at 94 °C for 15 min, 30 cycles of 94 °C for 30 s, 57 °C for 1 min, 72 °C for 1 min, followed by a final extension at 72 °C for 10 min.

### 4.5. Whole-Genome Sequencing

Whole-genome sequencing was performed with the Illumina MiSeq sequencer (Illumina, San Diego, CA, USA). The extracted DNA was quantified using a Qubit double-strand (ds) high-sensitivity (HS) kit (Thermo Fisher Scientific, Waltham, MA, USA). A DNA concentration between 100 and 500 ng in 30 µL of the extract was used as starting material for library preparation. Libraries were prepared according to the manufacturer’s instruction using the Nextera DNA Flex Library preparation kit (Illumina, San Diego, CA, USA). Amplified libraries were quantified with the 2100 bioanalyzer system (Agilent, Santa Clara, CA, USA) and subsequently by quantitative PCR (qPCR) using the Kapa Sybr Fast qPCR kit (Kapa Biosystems, Wilmington, MA, USA). Using the fragment sizes generated by the bioanalyzer and the concentration of individual libraries from the qPCR, the libraries were normalized, pooled, and loaded into the MiSeq.The MiSeq sequencer generated 250 paired-end reads with barcoding.

The raw fastq files were trimmed using BBDuk trimmer (v 1.0) (http://sourceforge.net/projects/bbmap/) at a Phred quality score ≥20. An in-house pipeline by US Army Medical Research Institute for Infectious Diseases (USAMRIID) was used to blast the trimmed reads to the NCBI non-redundant database. The best-matching sequences were selected and used as references to perform reference-based assembly. Assembly was done using Geneious Prime (v 2019.2) (www.geneious.com) while maintaining default settings with a minimum of 3 × read-depth coverage required for consensus calling.

The fasta files generated were then uploaded in the Center for Genomic Epidemiology webpage (https://cge.cbs.dtu.dk/services/ (accessed on 4 June 2019)) to determine the *spa* types, multi-locus sequence types, resistance, and virulence genes present. For phylogenetic analysis, whole-genome alignment was done using Mauve (v 1.1.1) [46]. The maximum likelihood phylogeny of *S. aureus* isolates was constructed based on the GTR GAMMA nucleotide substitution model [47] using RAxML (v 4.0) implemented in Geneious Prime (v 2020.0.4) (www.geneious.com).

### 4.6. Data Analysis

Demographic data and laboratory results were entered into Access 2007 and exported to STATA software version 13 for analysis. Descriptive statistical analysis was conducted and associations were determined using Fisher’s exact test with significant *p*-values < 0.05.

## 5. Conclusions

The epidemiology of *S. aureus* SSIs remains a public health concern in hospitals in Ghana. Novel molecular methods were utilized in Ghana to identify and characterize multi-drug-resistant *S. aureus* clones, including the pandemic *pvl*-positive ST152-t355 MRSA. These findings suggest that continuous surveillance is required to monitor the spread and resistance trends of these and other clones in hospital settings in Ghana and within the West African region.

## 6. Limitations

The study was limited by the number of *S. aureus* isolates, and it did not follow subjects prospectively to assess clinical outcomes. Additional data will allow an assessment of antimicrobial resistance over time and will also detect the emergence of resistant clones.

## Figures and Tables

**Table 1:** Demographic characteristics of patients enrolled at the two hospitals.

Characteristics	37 Military Hospital N = 56 n%)	Korle Bu Teaching Hospital N = 56 n(%)	Total N = 112 n(%)
*Gender*			
Male	28(50.0)	6(10.7)	34(30.4)
Female	28(50.0)	50(89.3)	78(69.6)
*Age*			
≤13 years	1(1.8)	6(10.7)	7(6.3)
>13 years	55(98.2)	50(89.3)	105(93.8)
*Department/ward*			
General surgery	20(35.7)	−	
Trauma and Surgical emergency unit	10(17.9)	−	
Surgical OPD	16(28.6)	−	
Obstetrics and Gynecology	10(17.9)	5(8.9)	
Maternity	−	38(67.9)	
Neurosurgery	−	2(3.6)	
Pediatric Unit	−	6(10.7)	
Surgical Unit	−	3(5.4)	
Orthopedic ward	−	2(3.6)	
*S. aureus positivity*	8(14.3)	5(8.9)	13(11.6)

**Table 2:** Sources and characteristics *S. aureus* isolates.

Hospital	Ward	Age	Gender	Operation Type	Antibiotic After Operation	POH	MRSA/MSSA	Antibiotic Resistance	*Spa type*/ST	SCC*mec* Type	Resistant Genes	Virulence Genes
37-MH	TSE	25	Male	Incision and drainage	Amoxicillin clavulanic acid	6days	MRSA	cef+ tet	t355(ST152)	IVa (2B)	*blaZ, tet(K), cat(pC221), mecA*	*hlgA, hlgB, lukS-PV, lukF-PV*
37-MH	GS	57	Female	Debridement of right foot	Ciprofloxacin	9days	MRSA	cef+ cli+ ery	t355(ST152)	IVa (2B)	*blaZ, erm(C), mecA*	*hlgA, hlgB, lukS-PV, lukF-PV, scn*
37-MH	GS	57	Male	Incision and drainage of Pus	Clindamycin	12days	MRSA	cef+ery	t355(ST152)	IVa (2B)	*cat(pC221), mecA*	*hlgA, hlgB, lukS-PV, lukF-PV, scn*
37-MH	SOPD	48	Male	Herniotomy	Ciprofloxacin	7days	MRSA	cef+tet+cli+ery	t002(ST5)	Vc	*blaZ, tet(K), Inu(A), mecA*	*hlgA, hlgB, hlgC, lukD, sei, sem, sen, seo, sep, scn, sak, aur, splA, splB*
37-MH	GS	49	Female	Incision and Drainage	Amoxicillin clavulanic acid, Cefuroxime	6days	MSSA	−	t442(ST5)	N/A	*blaZ*	*hlgA, hlgB, hlgC, lukD, lukE, lukS-PV, lukF-PV sea, seb, sei, sem, sen, seo, scn, sak, aur, splA*
37-MH	SOPD	41	Female	Open reduction internal fixation	Metronidazole	30days	MSSA	tet+cli	t127(ST1)	N/A	*blaZ, tet(K)*	*hlgA, hlgB, hlgC, lukE, sea, sek, tst, scn, sak, aur, splA, splB*
37-MH	SOPD	51	Male	Hernia Repair	No medication	5days	MSSA	tet+sxt+ery	t084(ST3249)	N/A	*blaZ, tet(K), dfrG*	*hlgA, hlgB, lukS-PV, lukF-PV, scn*
37-MH	GS	68	Male	Appendectomy	No medication	1day	MSSA	ery	t3194(ST30)	N/A	*blaZ, dfrG*	*hlgA, hlgB, hlgC, sei, seu, scn, aur, splE*
KBTH	MT	30	Female	Emergency Caesarean section	Amoxicillin clavulanic acid, Metronidazole	1day	MSSA	tet+ery	t355(ST152)	N/A	*blaZ, tet(K), Inu(A), aadD*	*hlgA, hlgB, lukS-PV, lukF-PV, scn*
KBTH	MT	32	Female	Elective Caesarean section	Gentamicin, Clindamycin	13 days	MSSA	tet	t355(ST152)	N/A	*blaZ, tet(K)*	*hlgA, hlgB, scn*
KBTH	MT	36	Female	Caesarean section	Amoxicillin clavulanic acid, Metronidazole	4days	MSSA	−	Unknown spa type (ST3249)	N/A	*blaZ*	*hlgB, lukS-PV, lukF-PV, scn*
KBTH	MT	34	Female	Caesarean section	Clindamycin	1days	MSSA	−	t4019(ST152)	N/A	*blaZ*	*hlgA, hlgB, lukS-PV, lukF-PV, scn*
KBTH	GS	15	Female	2^nd^ Stage Debulking	Cefuroxime	13days	MSSA	−	t002(ST5)	N/A	*blaZ*	*hlgA, hlgB, hlgC, lukD, sei, sem, sen, seo, sep, scn, sak, aur, splA, splB*

Abbreviations: cef: cefoxitin, tet: tetracycline, cli: clindamycin, ery: erythromycin, sxt: co-trimoxazole, 37-MH: 37 Military Hospital, KBTH: Korle-Bu Teaching Hospital. TSE: Trauma and Surgical Emergency; GS: General Surgery; SOPD: Surgical Outpatient Department; MT: Maternity; ST: sequence type; POH: period of hospitalization.

**Table 3:** Summary of characteristics of whole-genome and assembly properties of *S. aureus* isolates.

Isolate ID	No. of Reads	G+C Content (%)	Mean Coverage (X)	No. of Predicted Coding Sequences	No. of Predicted RNAs	GenBank Accession No.
**GHA1**	2,512,041	32.8	441	2699	82	CP043923.1
**GHA2**	2,096,320	32.9	379	2683	82	CP043922.1
**GHA3**	1,159,656	32.8	208	2675	81	CP043921.1
**GHA4**	133,790	32.9	24	2694	69	CP043920.1
**GHA5**	95,977	33.0	17	2612	77	CP043919.1
**GHA6**	3,080,479	32.8	554	2649	81	CP043918.1
**GHA7**	2,947,151	32.6	551	2564	56	CP043917.1
**GHA8**	3,412,807	32.8	612	2716	64	CP043916.1
**GHA9**	397,799	32.7	74	2632	58	CP043915.1
**GHA10**	1,274,423	32.9	233	2651	82	CP043914.1
**GHA11**	982,047	32.9	175	2653	80	CP043913.1
**GHA12**	891,432	32.9	166	2534	82	CP043912.1
**GHA13**	1,882,838	32.7	352	2628	57	CP043911.1

**Table 4:** Primer sequences used in the study.

Primer	Primer Sequences	Description	References
*spa*	F: 5′-TAAAGACGATCCTTCGGTGAGC-3′R: 5′-CAGCAGTAGTGCCGTTTGCTT-3′	To detect the *spa* gene (*S. aureus* specific)	[45]
*pvl*	F: 5′-GCTGGACAAAACTTCTTGGAATAT-3′R: 5′-GATAGGACACCAATAAATTCTGGATTG-3′	To detect Panton-Valentine leukocidin (virulence factor)	[45]
*mecA*	F: 5′-TCCAGATTACA ACTTCACCAGG-3′R: 5′-CCACTTCATATCTTGTAACG-3′	To detect methicillin resistance due to the *mecA* gene	[45]

## Data Availability

The data used and/or analyzed during the current study are available from the corresponding author on reasonable request.
